# Walking elicits muscle functional changes in the pectoral fin of *Polypterus senegalus*

**DOI:** 10.1242/jeb.250474

**Published:** 2025-11-06

**Authors:** Lisha Liang, Linfang Han, Misha Dhuper, Keegan Lutek, Emily M. Standen

**Affiliations:** ^1^ University of Ottawa, Department of Biology, 30 Marie-Curie Private, Ottawa, ON K1N 9A7, Canada; ^2^ Villanova University, Department of Biology, 800 Lancaster Avenue, Villanova, PA 19085, USA

**Keywords:** Muscle function, Electromyography, Amphibious locomotion, Tissue plasticity

## Abstract

Amphibious fishes are often used as models to help understand how early aquatic vertebrates overcame the mechanical and physical challenges posed by a terrestrial environment. The differences in posture and loading required on land affect fin function and, over longer durations, may elicit changes in muscle and tissue composition, altering performance. How the motor control patterns of fin muscles change in a walking gait is not known but may help explain the changes in bone remodelling and muscle fibre type that occur in *Polypterus senegalus*, when exercised or kept in a terrestrial environment. This study quantified instantaneous motor activation changes in all four fin muscle groups involved in the terrestrial walking gait in *P. senegalus*. We discovered that increases in the operating length of muscles and in the velocity of contraction (and subsequent expected rate of force production), prolonged muscle use, and changes in eccentric and antagonistic co-contraction occur in muscles when used for walking compared with swimming. It appears that subtle changes in motor patterns between swimming and walking can elicit large changes in functional performance, which helps explain muscle remodelling seen in fish that spend long periods of time in terrestrial environments.

## Teaser

**Summary:** In *Polypterus senegalus*, fin position, fin loading and muscle activation timing change during the stroke cycle when walking, possibly explaining muscle fibre-type changes seen in fish raised on land.

## INTRODUCTION

In recent years, various patterns of locomotion in amphibious fishes have been used to infer mechanical and physiological challenges that had to be overcome as early vertebrates evolved to live in terrestrial environments. *Polypterus senegalus*, one species that has emerged as a model in this area of research, shows changes in bone and muscle morphology when exposed for prolonged durations to land (Du and Standen, 2017; Standen et al., 2014). Although the muscle tissue changes from aerobic to anaerobic fibres have been well documented (Du and Standen, 2017), the exact mechanism driving these changes is unknown. The focus of this study was to take a closer look at how *P. senegalus* changes fin muscle use when moving on land. We quantified changes in muscle effort and activation timing relative to fin motion during swimming and walking in animals that live an aquatic lifestyle. Comparing instantaneous changes in kinematics and muscle activation between locomotor modes can help to identify mechanical and physiological mechanisms associated with terrestrial locomotion that could drive the tissue plasticity that has been seen in these animals when held in terrestrial environments over the longer term.

In water, fishes are supported by buoyancy but must overcome drag to generate forward movement. On land, fish must support their own body weight and overcome friction between their body and the substrate. These changes in physical environment mean that terrestrial locomotion tends to have a higher cost of transport and requires greater vertical forces than aquatic locomotion (Kok et al., 1998; Schmidt-Nielsen, 1972). As a result, amphibious fish on land require greater muscle effort to generate forward movement (Foster et al., 2018; Gillis, 2000; Perlman and Ashley-Ross, 2016). In addition, amphibious fish alter their behaviour, moving their bodies in different ways and changing musculoskeletal loading when on land (Lutek et al., 2022a; Perlman and Ashley-Ross, 2016; Standen et al., 2014; Wright and Turko, 2016). Terrestrial locomotion therefore increases force demands and changes force distribution on the neuromuscular system of amphibious fishes. The goal of this study was to compare the muscle activation and kinematic output of fins between walking and swimming to formulate hypotheses about how these changed force environments may be contributing to documented muscle fibre changes.

**Fig. 1.**
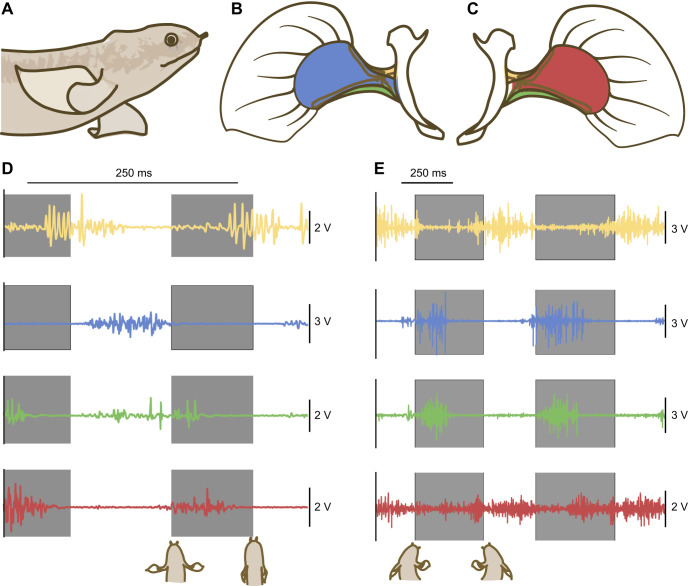
**Example traces of pectoral fin muscle activity during swimming and walking in a representative *Polypterus senegalus*.** (A) Lateral view of the right pectoral fin during the swing phase of a walking step. (B) Lateral view of the right pectoral fin with skin cut away to show the abductor (Abd; blue), coracometapterygialis (Cmt; green) and zonopropterygialis (Zpt; yellow). (C) Medial view of the right pectoral fin with the skin cut away to show the Add (red), Cmt (green) and Zpt (yellow). (D,E) Pectoral fin muscle activity during routine swimming (D) and walking (E) in one individual. Colours match those in B and C. The grey boxes represent the propulsive phase of the stroke; the unshaded portions represent the recovery phase of the stroke.

*Polypterus senegalus*, the extant ray-finned fish closest to the actinopterygian–sarcopterygian common ancestor, is predominantly aquatic and has the capacity to breathe air and locomote overland (Standen et al., 2014)*. Polypterus senegalus* have a complex pectoral fin musculature consisting of several muscles, categorized into four groups: zonopropterygialis (Zpt), abductor (Abd; superficialis and profundus combined), coracometapterygialis (Cmt; Cmt I and II combined) and adductor (Add; superficialis and profundus combined) (Fig. 1; Wilhelm et al., 2015). As in most other vertebrates, these muscles are made of at least two types of muscle fibres (Du and Standen, 2017). ‘Slow’ muscle fibres are confined to the periphery of each pectoral fin muscle and likely correspond to ‘red’ fibres, which rely on ATP produced aerobically, contract slowly and generate relatively low force (Herbison et al., 1982; Schiaffino and Reggiani, 2011). ‘Fast’ muscle fibres are found in the interior of each muscle and likely correspond to ‘white’ fibres, which rely on anaerobically produced ATP, contract quickly and produce relatively high force (Herbison et al., 1982; Jayne and Lauder, 1993, 1994). Muscle tissue and resulting fibre type is plastic, responding to differences in how muscles are used. *Polypterus senegalus* fin musculature has been shown to be plastic in response to prolonged exposure to terrestrial environments (Du and Standen, 2017). In this study, we quantified the changes in muscle activation and kinematics that occur between swimming and walking in the short term, to help explain previously seen changes in muscle fibres after long-term exposure to land.

*Polypterus senegalus* have distinct locomotor kinematics when they swim and walk (Standen et al., 2014). During swimming, *P. senegalus* rely on synchronized pectoral fin oscillations to generate thrust. During walking, *P. senegalus* lift their heads by planting their pectoral fins in a contralateral pattern one after the other and use large body and tail oscillations to push themselves forward over the planted fin (Fig. 6). Current evidence suggests that pectoral fin Add muscles generate an additional contraction burst during the step cycle, which co-activates with the Abd muscle, providing a stabilizing force (Foster et al., 2018). This stabilizing co-contraction, in combination with gravitational loading of the body as it is launched over the fin, likely maintains a fin position that results in eccentric loading of some muscle groups. Walking bouts may, therefore, represent prolonged exercise, muscle overstretching and potential strength training – all of which lead to active lengthening, which is associated with muscle damage in other vertebrate systems (Appell et al., 1992; Chargé and Rudnicki, 2004; Coso et al., 2012) and is known to initiate the transition from red to white fibres (McClelland, 2012). Minimal activation data on the Add and Abd muscles of *P. senegalus* exist (Foster et al., 2018); however, a detailed description of how muscle activation relates to kinematic function across all fin musculature is not clear and necessitates further exploration to substantiate hypotheses about how changing fin function can lead to the previously quantified muscle fibre-type plasticity that occurs after terrestrial acclimation.

The general goal of this study was to answer the following question: ‘Can changes in muscle activation patterns relative to kinematic changes explain changes in muscle fibre type seen in the pectoral fins of *P. senegalus* that are exposed to terrestrial environments?’. We hypothesized that novel muscle functional performance required during walking triggers the plastic change in muscle fibre type seen in fish exposed to terrestrial environments. Specifically, we predicted that walking behaviour causes major changes in fin use, increasing the length at which muscles must operate, increasing eccentric contraction episodes and prolonging active muscle loading. In addition, we predicted that muscle shortening velocity and apparent force loading will increase during walking. As shortening velocity increases, force production decreases; therefore, these requirements contradict the known skeletal muscle force–velocity properties putting extra demand on muscle fibres. We tested these predictions using electromyography (EMG) and kinematic analysis to relate muscle activity to fin position and to estimate changes in muscle length during contraction, muscle effort and antagonistic action between muscle groups. We also used a preliminary quantification of pectoral fin muscle membrane permeability as a proxy for muscle damage after short bouts of walking to gain some understanding of which muscles are engaged in strenuous activity during walking. Understanding how swimming and walking muscle activation and kinematics differ in fish from an aquatic environment may help reveal mechanisms that lead to changes in muscle use and muscle fibre damage in the short term. These same short-term changes may lead to the tissue plasticity seen when amphibious fishes are exposed to land over the long term.

## MATERIALS AND METHODS

### Subjects

Four adult *Polypterus senegalus* Cuvier 1829 raised in aquatic environments [mass±s.e.m., 14.20±1.08 g; body length (BL)±s.e.m., 137.3±5 mm] were used for the muscle activity experiments (Mirdo Importations Canada, Montreal, QC, Canada). Two additional groups of fish of three size classes were used to estimate walking-induced muscle damage using Evan's Blue dye. The first group of fish included four smaller adult *P. senegalus* [mass, 2.97 g; total length (BL), 78.75 mm; original values are not available so no s.e.m. could be calculated; AQUAlity, Toronto, ON, Canada]. A second group of eight larger fish were tested to size match the fish used in the muscle activity portion of the study (two medium fish, mass±s.e.m., 4.30±0.025 g; BL±s.e.m., 90.00±5.0 mm; six larger fish, mass±s.e.m., 9.37±0.18 g; BL±s.e.m., 117.67±2.06 mm; Tropical Inc., Montreal, PQ, Canada). All fish were housed in individual flow through tanks on a 12 h:12 h light: dark cycle. Fish for the muscle activity experiments were starved for 24 h before surgery to minimize the effect of undigested food on anaesthesia (Foster et al., 2018). The sex of all fish was unknown. Sample sizes were determined based on previous experiments (Lutek et al., 2022b). All experimental procedures were in accordance with the University of Ottawa Animal Care and Use Protocols BL2069 and BL3625.

### Surgery

Before EMG electrode implantation surgery, fish were anaesthetized in buffered 200 mg l^−1^ MS-222 solution (tricaine methanesulfonate; Sigma-Aldrich, St Louis, MO, USA) until breathing slowed and fish were unresponsive to tail pinching. Hook electrodes made from bi-filament stainless steel electrode wire (0.051 mm diameter; California Fine Wire Company, Grover Beach, CA, USA) were placed percutaneously into four muscle groups of the pectoral fin – Abd, Add, Cmt and Zpt – using sharp 30-guage needles. Each fish was then allowed to recover in fish housing water until they regained consciousness and began voluntary swimming. After behavioural trials were completed, fish were euthanized with an overdose of buffered MS-222 (417 mg l^−1^), and electrode position was confirmed under a dissection microscope.

### Experimental procedure

Recovered fish were gently encouraged to perform steady swimming and then walking in a plexiglass tank (33×76×15 cm; width×length×height). Swimming trials were recorded with 5 cm of fish housing water. Walking trials were recorded without any water. Fish were given 1–2 h break between the two behaviours to ensure that there were no effects of fatigue. For both behaviours, we recorded steady bouts of locomotion that consisted of at least three consecutive strokes until we had a total of at least 10 strokes for each fish. All trials were filmed at 500 frames s^−1^ from the bottom and the side using two Photron Fastcam Mini UX100 cameras (Photron USA, San Diego, CA, USA). Cameras were calibrated in MATLAB (version R2020a; MathWorks, Natick, MA, USA) using a 36-point calibration object in custom DLTdv8 software (Hedrick, 2008).

Muscle activity recordings and high-speed videos were synchronized by an external trigger. Muscle activity signals were collected at 10 kHz using an AD Instruments PowerLab 16/35 data acquisition system (ADInstruments, Colorado Springs, CO, USA). The signals were amplified 5000 times and filtered with a 60 Hz notch filter using GRASS P511 AC amplifiers (Natus Neurology, Warwick, RI, USA). Movement artifacts and background noise were filtered out with a bandpass filter (40–4000 Hz) in MATLAB using custom code.

### Kinematics analysis

Three-dimensional trajectories of the nose tip, caudal fin tip, tip of the rays of the right pectoral fin and the dorsal edge of the right pectoral fin base were digitized using DLTdv8 (Hedrick, 2008). From these points, variables that quantified the magnitude and frequency of kinematic movements were calculated. Speed over ground was calculated as the distance travelled along the path of the fish over time (BL s^−1^). Swing distances for the caudal and pectoral fin tips were calculated as the total distance travelled in the *x*-*y* plane throughout a complete oscillation cycle for the fin. Pectoral fin elevation maximum, minimum and range were calculated based on the vertical movement of the pectoral fin tip (BL). Larger values indicated that the fin was positioned more dorsally. Pectoral fin adduction angle maximum, minimum and range were defined by the angle between the nose tip, the dorsal edge of the pectoral fin base and the pectoral fin tip in the *x*-*y* plane. Larger values indicated that the pectoral fin was more adducted. Pectoral fin angular velocity was calculated for abduction and for adduction (deg s^−1^). The mean of the top 5% of angular velocities was used to represent maximum pectoral fin angular velocity. The mean of the top 20% of values was used to represent routine pectoral fin angular velocity. Negative and positive values of pectoral fin angular velocity represent pectoral fin abduction and pectoral fin adduction, respectively. For walking only, we also calculated swing distance of the nose (BL; calculated similarly to tail fins above).

We defined the start and middle of the swimming stroke cycle by visually identifying the change in the direction of fin motion associated with the start of the power stroke (cycle start) and the start of the recovery stroke (mid cycle) in the open-source image analysis software FIJI (Schindelin et al., 2012). We defined the start and middle of the walking stroke cycle by visually identifying the beginning of stance (cycle start; when the fin was first loaded on the ground) and the beginning of swing (mid-cycle; when the fin ceased to be loaded). We refer to the propulsive and recovery phases, where the propulsive phase occurs during adduction in swimming and stance in walking, and the recovery phase is during abduction in swimming and swing during walking. We present the following kinematic timings in polar coordinates (deg) relative to the start of propulsion and the start of recovery within the stroke cycle: maximum and minimum nose elevation, maximum and minimum fin elevation, and maximum and minimum fin adduction angle.

### Electromyography analysis

All EMG variables were calculated after the background signal noise (the mean signal from an inactive portion of the EMG trace) was subtracted from the raw EMG data; this centred the EMG data around zero and is referred to as the centred EMG data. Onset and offset timings were identified by a procedure similar to that published by Ozgünen et al. (2010), which uses a threshold against the root mean square of a muscle activity signal to identify when a muscle is active. First, centred EMG data were denoised (hereafter denoised EMG data) using an empirical Bayesian method (‘wdenoise’ function in MATLAB). We maximized the amount of noise removed from the signal using a mean threshold rule within the function, applying a noise level-dependent estimate of the noise variance. We then identified muscle activity onsets and offsets using the moving root mean square envelope (400-sample window, corresponding to 40 ms) of the denoised EMG data. These muscle timings were defined as the times when the envelope crossed 25% of the mean of the root mean square envelope. From this preliminary set of timings, we removed any instances in which the burst required the muscle to turn on and off at a rate higher than 100 Hz to remove bursts that were likely outside the realm of physiological possibility for fish body muscle (for frequencies that achieve tetanus in zebrafish skeletal muscle, see Idoux et al., 2022).

For each occurrence that the muscle was turned on, we calculated burst duration (s), EMG duty factor (% stroke cycle), rectified integrated area (RIA; % theoretical maximum) and maximum burst amplitude (% maximum EMG signal) from the centred EMG data similarly to previous investigations (Foster et al., 2018; Lutek and Standen, 2021; Lutek et al., 2022b). Because we observed multiple bursts (one to six, depending on the muscle) within a single kinematic cycle, we summed cumulative variables across the full cycle as well as for just the propulsive phase and just the recovery phase for each cycle. Maximum burst amplitude was calculated as the mean of the top 5% of signals within a single muscle burst. For full cycle, propulsive or recovery phases that had multiple bursts, the mean of the top 5% of signals across all bursts was used. To permit comparison between individual fish and electrodes, each maximum burst amplitude was then divided by the highest maximum burst amplitude recorded on that channel for the fish and reported as % maximum burst amplitude. This maximum activation value was also used to calculate a theoretical maximum RIA for each burst, the product of this maximum activation value and burst duration. RIA values are presented as a percentage of this theoretical maximum to permit comparisons between individuals and electrodes. Onset and offset timings are expressed in polar coordinates (deg) relative to the pectoral fin stroke.

### Muscle damage experiments

The methodology for these experiments was adapted from Hamer et al. (2002), with slight modifications to account for the slower metabolism of fish. Fish were anaesthetized using buffered 125 mg l^−1^ MS-222 (tricaine methanesulfonate; Sigma-Aldrich), injected intraperitoneally with 1% (volume/weight) Evans Blue dye and returned to fish housing water for 30 h. Treatment fish were then walked continuously to exhaustion (1–2 min). After walking, treatment fish were returned to fish water for 2 h. Treatment (Evans Blue dye injection, with walking) and control (Evans Blue dye injection, no walking) fish were then euthanized with an overdose of buffered MS-222 (417 mg l^−1^). The pectoral fins were dissected from the body, and the upper layers of skin and scales were removed. Samples were then embedded into moulds in optimal cutting temperature compound (Fisher Scientific, Hampton, NH, USA) and snap frozen using a typical isopentane and liquid nitrogen protocol (Meng et al., 2014). Samples were stored in a −80°C freezer until cryo-sectioning.

The frozen blocks were sectioned into 10 µm slices on a Leica CM 1850 or CM3050S cryostat (Leica, Wetzlar, Germany) at −21°C. Slides were dipped into cold acetone (−20°C) for 1 min or into room temperature acetone for 5 min and then air dried at room temperature (20–22°C). The sections were then dipped into a xylene substitute (Shandon) for sufficient time to mount with DPx (Sigma-Aldrich) and a glass coverslip. Slides were stored in the dark to minimize photobleaching.

Frozen sections were viewed by fluorescent microscopy on two different systems. The first set of fish were imaged using an Axiphot (Zeiss, Oberkochen, Germany) using a N2.1 green wavelength filter set, a bandpass filter of 515–560 nm and a low-pass filter of 590 nm. Images were acquired with a DP-70 Colour CCD camera (Olympus, Shinjuku City, Japan) at 10× and 20× magnifications. The second set of fish were imaged using an Axio Imager (Carl Zeiss Canada) and the Alexa Fluor 568 filter set (excitation, 577 nm; emission, 603 nm; Carl Zeiss Canada). Images were acquired with an Axiocam 705 momo (Carl Zeiss Canada). All images were collected at standardized exposure settings to permit comparisons and stored without any processing.

Cross-sectional images were analysed using ImageJ (National Institutes of Health, Bethesda, MD, USA). Fluorescence intensity was quantified by using the multi-point tool to determine the integrated density of fluorescence at the centre of all identifiable cells. Background fluorescence measurements were made at the darkest site adjacent to the muscle that contained no tissue, and this value was subtracted from the intensity at the centre of each cell. We then calculated a mean integrated density value for each muscle for analysis. Higher values of fluorescence indicated that more Evans Blue dye had infiltrated the cell.

Distance along the fin was measured from the distal tip of the fleshy fin lobe (100% fin length) until the fin muscle could no longer be clearly identified at the base of the fin (0% fin length).

### Statistical analyses

Linear mixed-effects (LME) models were conducted using the nlme package (https://CRAN.R-project.org/package=nlme) in R Studio (version 9.0; Boston, MA, USA) to evaluate differences in each linear variable between behaviours or treatments and across muscles, including corrections for unequal variance as appropriate. Models for kinematic variables had behaviour as a fixed effect. Models for EMG variables had both behaviour and muscle as fixed effects. In all cases, we modelled a random intercept for each individual. When necessary, multiple comparisons were computed on the linear EMG variables to determine differences between behaviours across muscles. Multiple comparison *P*-values were Bonferroni corrected based on the number of comparisons to reduce type I error. LME results are reported as ANOVA-like tables. For the Evans Blue dye experiments, we constructed simple linear models for each size group separately that had distance along the pectoral fin and treatment as predictors, and Cohen's *d* was used to estimate the magnitude of treatment effects. All linear statistics were performed using custom code. The assumptions of normality and homoskedasticity of residuals were assessed visually and statistically. Mean values for linear variables are presented as estimated marginal means±s.e.m. (emmeans package; https://cran.r-project.org/web/packages/emmeans/index.html) unless stated otherwise.

Polar variables were analysed using standard circular statistics (Zar, 1999) as in Standen et al. (2014), utilizing custom MATLAB code. Variables were first tested for a von Mises distribution and equal variation using a Kuiper test (Zar, 1999). If the data had a von Mises distribution, we tested whether data occurred at a consistent point in the kinematic cycle using Rayleigh's test. If the data did not have a von Mises distribution, we used the Hermans–Rasson test as an alternative to Rayleigh's test (Landler et al., 2019). Finally, we tested for differences in the mean timing of each variable between swimming and walking. Data with a von Mises distribution were tested for this difference using the Watson-Williams test (Batschelet, 1981; Zar, 1999). Data that did not have a von Mises distribution were tested for a difference in mean timing using the non-parametric Watson test as an alternative. These differences in mean timing were tested only when a variable had a consistent timing in the stroke cycle for both swimming and walking. Mean polar timings are presented as angular mean±angular variance.

## RESULTS

### Kinematic differences between swimming and walking

**Fig. 2.**
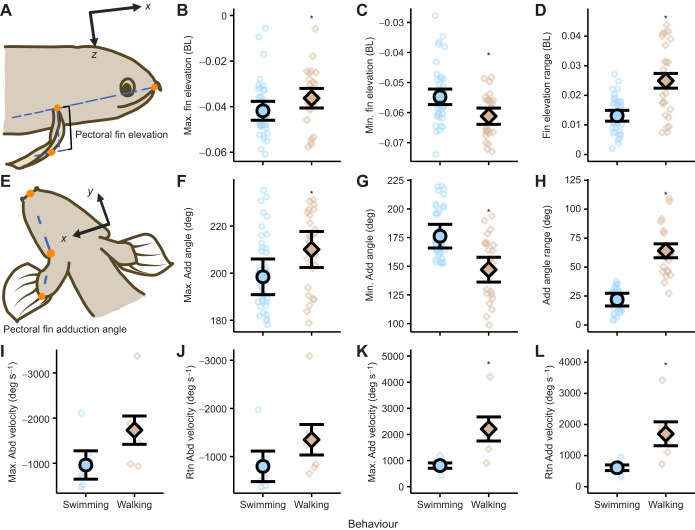
**Differences in pectoral fin kinematics between swimming and walking in *P. senegalus*.** Swimming and walking pectoral fin kinematics were recorded in *P. senegalus* (*N*=4) following muscle electrode implantation. (A,E) Illustrations of how pectoral fin elevation (A) and pectoral fin adduction angle (E) were measured. (B–D,F–H) Pectoral fin elevation (B–D) and adduction angle (F–H) changes between behaviours suggest increased range of pectoral fin motion during walking. (I,J) Pectoral fin abduction speed during swimming and walking does not change significantly. (K,L) Pectoral adductor speed increases during walking. Small points are trial means. Large points and error bars are the estimated marginal mean±s.e.m. for each behaviour. Asterisks denote a statistically significant difference between walking and swimming (linear mixed-effects model; *P*<0.05). Abd, abduction; Add, adduction; BL, body length; Max., maximum (for I and K, mean of the top 5% of values); Min., minimum; Rtn, routine (for J and L, mean of the top 20% of values).

Speed over ground did not differ between swimming and walking (Table 1). Swing distance was significantly higher during walking compared with swimming for the tip of the caudal and pectoral fins (Table 1). Maximum fin elevation was higher, and minimum fin elevation was lower, and therefore the range of fin elevation was larger for walking than for swimming (Fig. 2A–D, Table 1). During walking, maximum adduction angle was larger and minimum adduction angle was smaller than during swimming (Fig. 2E-G, Table 1), making adduction angle range larger during walking than during swimming (Fig. 2H, Table 1). Maximum and routine pectoral fin angular velocity during abduction did not differ between behaviours (Fig. 2I,J, Table 1); however, maximum and routine angular velocity during adduction was higher during walking than during swimming (Fig. 2K,L, Table 1).

**Table 1.** Behaviour during swimming and walking in *Polypterus senegalus*

Variable	*F*	d.f.	*P*	*R* ^2^_m_	*R* ^2^_c_	emmean±s.e.m.	
						Swiming	Walking
Speed over ground (BL s^−1^)	0.07	1,7	0.79	0.01	0.28	0.53±0.082	0.56±0.082
Caudal fin swing distance (BL)	59.64	1,20	**<0.001**	0.71	0.71	0.80±0.24	2.98±0.15
Pectoral fin swing distance (BL)	65.05	1,60	**<0.001**	0.82	0.83	0.24±0.02	0.65±0.05
Maximum pectoral fin elevation (BL)	5.26	1,66	**0.02**	0.04	0.41	−0.042±0.004	−0.036±0.004
Minimum pectoral fin elevation (BL)	12.31	1,66	**<0.001**	0.11	0.35	−0.055±0.003	−0.061±0.003
Pectoral fin elevation range (BL)	32.44	1,66	**<0.001**	0.46	0.60	0.013±0.002	0.025±0.003
Maximum pectoral fin adduction (deg)	28.49	1,67	**<0.001**	0.10	0.76	198±7.6	210±7.6
Minimum pectoral fin adduction (deg)	61.35	1,67	**<0.001**	0.30	0.90	176±10.3	147±10.8
Pectoral fin adduction range (deg)	61.43	1,7	**<0.001**	0.72	0.94	21.9±5.5	64.1±6.0
Maximum abduction velocity (deg s^−1^)	3.03	1,7	0.13	0.22	0.22	−960±314	−1733±314
Routine abduction velocity (deg s^−1^)	1.53	1,7	0.26	0.12	0.12	−798±316	−1351±316
Maximum adduction velocity (deg s^−1^)	8.8	1,7	**0.02**	0.89	0.89	808±103	2211±462
Routine adduction velocity (deg s^−1^)	7.67	1,7	**0.03**	0.87	0.87	608±91	1701±384
Nose swing distance (BL)	−	−	−	−	−	−	1.10±0.04*

ANOVA-type results of linear mixed-effects models testing differences in kinematics between swimming and walking. Statistically significant *P*-values are in bold. BL, body length; emmean, estimated marginal mean; *R*^2^_m_, marginal r-squared (fixed effects only); *R*^2^_c_, conditional r-squared (whole model); *, arithmetic mean.

**Fig. 3.**
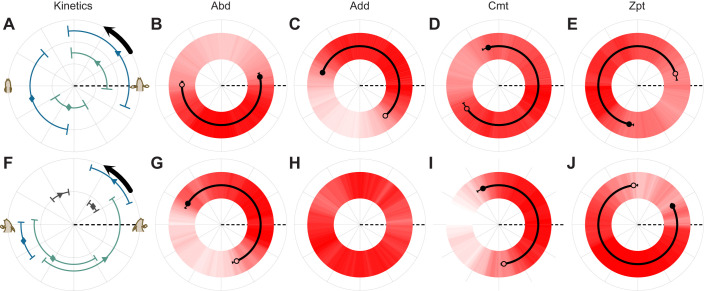
**Kinematic and muscle activity timing differences between swimming and walking in *P. senegalus*.** (A–J) Swimming (A–E) and walking (F–J) kinematics and muscle activity were recorded following electrode implantation in *P. senegalus* (*N*=4). Data are shown as polar coordinates relative to the right pectoral fin cycle. For kinematics (A,F), grey, green and blue points represent the mean timing of nose elevation, fin elevation and fin adduction, respectively (diamonds, maximum values; triangles, minimum values). Means and error bars represent angular mean and variance, respectively, and are shown only for variables that are non-uniformly distributed around the stroke (i.e. statistically predictable timing). For muscle activity [Abd (B,G), Add (C,H), Cmt (D,I), Zpt (E,J)], a relative count of how many times each muscle was active at a given point in the kinematic cycle is shown in red, with darker shades indicating that the muscle was more often active. Mean muscle activity onset (open circles) and offset (filled circles) timings (±angular s.e.m.) are overlaid on this representation of muscle activity for data that are non-uniformly distributed about the circle (note that some s.e.m. are smaller than the size of the point depicting the mean). Onset and offset timings are non-uniformly distributed about the pectoral fin stroke (i.e. statistically predictable timing; B–E,G,I,J; Hermans–Rasson test; *P*<0.05) for all muscles during swimming and all muscles except the Add (H) during walking. The start of the stroke cycle is at 0 deg (dashed lines), and the cycle proceeds counter clockwise around the circle (black arrows in A and F). Abd, abductor; Add, adductor; Cmt, coracometapterygialis; Zpt, zonopropterygialis.

Maximum and minimum adduction timing did not differ between swimming and walking (Fig. 3A,F, Table 2, Table S1). Maximum fin adduction occurred just after the beginning of recovery, and minimum fin adduction occurred just after the beginning of the propulsive portion of the stroke. Maximum pectoral fin elevation timing was not significantly different between swimming and walking (Fig. 3A,F, Table 2, Table S1). Minimum pectoral fin elevation timing was consistently just after the beginning of the propulsive phase during swimming but showed no consistent timing during walking (Fig. 3A,F, Table 2, Table S1). Maximum nose elevation during walking consistently occurred just after the beginning of propulsion, and minimum nose elevation consistently occurred before the end of propulsion (Fig. 3A,F, Table 2, Table S1).

**Table 2.** Differences in angular mean timing between swimming and walking in *P. senegalus*

Variable	Muscle	*F*	d.f.	*P*	amean±a.var.	
					Swimming	Walking
Maximum pectoral fin adduction (deg)	−	0.01	1,71	0.94	256±45	238±65
Minimum pectoral fin adduction (deg)	−	0.51	1,68	0.48	45±50	312±82
Maximum pectoral fin elevation (deg)	−	1.14	1,68	0.10	197±65	198±21
Minimum pectoral fin elevation (deg)	−	−	−	−	37±59	−
Maximum nose elevation (deg)	−	−	−	−	n/a	43±12
Minimum nose elevation (deg)	−	−	−	−	n/a	115±16
Muscle activity onset (deg)	Abd	76.33	1111	**<0.001**	179±44	292±60
	Add	−	−	−	310±30	−
	Cmt	18.13	1121	**<0.001**	215±78	277±42
	Zpt	54.63	1134	**<0.001**	18±78	96±56
Muscle activity offset (deg)	Abd	79.59	1115	**<0.001**	13±47	148±73
	Add	−	−	−	161±28	−
	Cmt	0.05*	n/a	>0.99	105±76	114±90
	Zpt	108.52	1113	**<0.001**	257±57	28±39

Differences in angular mean timing were assessed only when the angular timing of each variable for both swimming and walking was non-uniform (as assessed by Rayleigh's test or Hermans–Rasson test; Table S1). If the data had a von Mises distribution and a mean resultant vector length >0.45, a Watson-Williams test was used; otherwise, a non-parametric Watson test was run (*; test statistic is *U*^2^, not *F*). Significant *P*-values are in bold. Angular mean (amean) and angular variance (a.var.) are only presented when either Rayleigh's test or the Hermans–Rasson test suggest that the variable is not distributed uniformly about the circle (Table S1). Abd, abductor; Add, adductor; Cmt, coracometapterygialis; n/a, not applicable; Zpt, zonopropterygialis.

### Muscle activity changes between swimming and walking

**Fig. 4.**
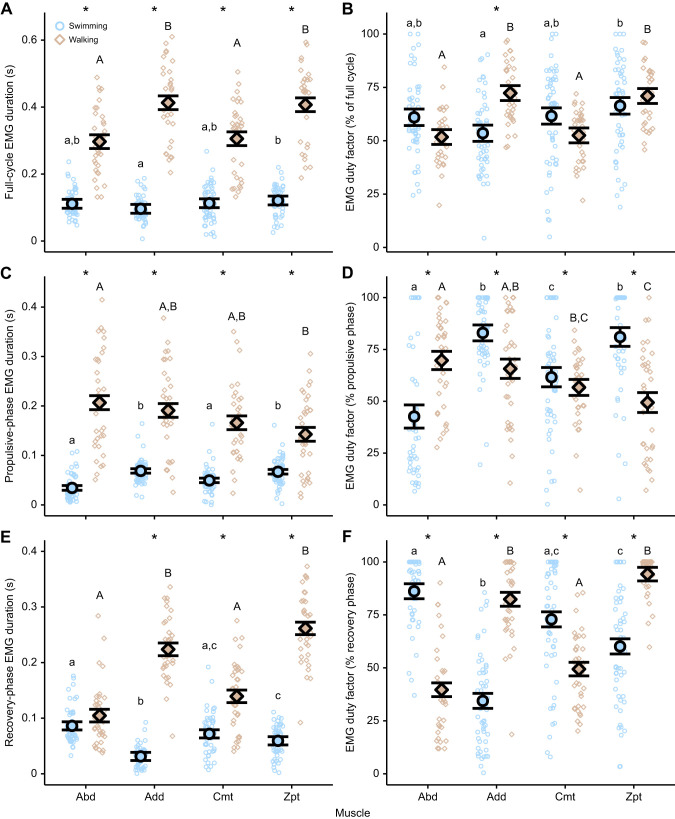
**Pectoral fin muscle activity duration (EMG duration and EMG duty factor) differences between swimming and walking *P. senegalus*.** Muscle activity was recorded from four muscle groups in the pectoral fin during routine swimming and walking in *P. senegalus* (*N*=4). (A,B) EMG duration and duty factor for the full stroke cycle. (C,D) EMG duration and duty factor for the propulsive-phase only. (E,F) EMG duration and duty factor for the recovery-phase only. Small symbols are values from each cycle or phase. Larger symbols and error bars are the estimated marginal mean±s.e.m. for each behaviour. Letters denote means that are statistically different within a behaviour (Bonferroni-corrected *post hoc* comparisons; *P*<0.05; lower-case letters are differences during swimming, upper-case letters are differences during walking). Asterisks denote a significant difference between behaviours within a muscle (Bonferroni-corrected *post hoc* comparisons; *P*<0.05). Abd, abductor; Add, adductor; Cmt, coracometapterygialis; EMG, electromyography; Zpt, zonopropterygialis.

All muscles had a higher full cycle muscle EMG activation duration (s) during walking than during swimming, primarily because a walking step is longer in duration than a swimming stroke (Fig. 4A, Table 3). This pattern generally held true when the propulsive and recovery phases were analysed independently, with the only exception being the Abd muscle, which had similar muscle activation during recovery for walking and swimming (Fig. 4C,E, Table 3). If muscle activation is calculated as the amount of time active per cycle (EMG duty factor), and we look at the entire stroke cycle, only the Add was higher during walking than during swimming (Fig. 4B, Table 3). Interestingly, when analysing by propulsion and recovery stroke phase independently, we saw significant differences in muscle behaviour between walking and swimming. During propulsion, Add, Cmt and Zpt muscles had a lower EMG duty factor, and the Abd had a higher EMG duty factor, during walking than during swimming (Fig. 4D, Table 3). In contrast, during recovery, these relationships were largely inverted (Fig. 4F, Table 3).

**Table 3.** Pectoral fin muscle activity during swimming and walking in *P. senegalus*

Variable	Fixed effect	*F*	d.f.	*P*	*R* ^2^_m_	*R* ^2^_c_	emmean±s.e.m.				
							Muscle	Swimming	Walking	*P*	*P*_bonf_
Full-cycle muscle activation duration (s)	Behav	113.82	1343	**<0.001**	0.88	0.91	Abd	0.111±0.013	0.297±0.021	<0.001	**<0.001**
	Muscle	3.91	3343	**0.009**			Add	0.097±0.013	0.413±0.021	<0.001	**<0.001**
	Behav×Muscle	14.49	3343	**<0.001**			Cmt	0.113±0.013	0.306±0.021	<0.001	**<0.001**
				** **			Zpt	0.121±0.013	0.407±0.021	<0.001	**<0.001**
Propulsion-phase muscle activation duration (s)	Behav	144.68	1343	**<0.001**	0.84	0.85	Abd	0.034±0.005	0.207±0.014	<0.001	**<0.001**
	Muscle	19.32	3343	**<0.001**			Add	0.069±0.005	0.191±0.014	<0.001	**<0.001**
	Behav×Muscle	7.76	3343	**<0.001**			Cmt	0.050±0.005	0.166±0.014	<0.001	**<0.001**
				** **			Zpt	0.067±0.005	0.143±0.014	<0.001	**<0.001**
Recovery-phase muscle activation duration (s)	Behav	3.27	1346	0.072	0.86	0.88	Abd	0.086±0.007	0.105±0.011	0.072	>0.99
	Muscle	39.30	3346	**<0.001**			Add	0.031±0.007	0.224±0.012	<0.001	**<0.001**
	Behav×Muscle	81.19	3346	**<0.001**			Cmt	0.072±0.007	0.140±0.011	<0.001	**<0.001**
				** **			Zpt	0.060±0.007	0.261±0.011	<0.001	**<0.001**
Muscle duty factor (% full cycle)	Behav	7.43	1343	**0.007**	0.11	0.17	Abd	61.1±3.9	51.8±3.5	0.007	0.11
	Muscle	4.10	3343	**0.007**			Add	53.5±3.8	72.3±3.5	<0.001	**<0.001**
	Behav×Muscle	16.14	3343	**<0.001**			Cmt	61.6±3.8	52.5±3.5	0.007	0.11
				** **			Zpt	66.4±3.9	71.0±3.5	0.18	>0.99
Muscle duty factor (% propulsive phase)	Behav	21.89	1343	**<0.001**	0.23	0.28	Abd	42.6±5.6	69.7±4.4	<0.001	**<0.001**
	Muscle	24.18	3343	**<0.001**			Add	83.0±3.8	65.7±4.7	<0.001	**0.002**
	Behav×Muscle	20.96	3343	**<0.001**			Cmt	61.6±4.7	56.7±3.9	0.26	>0.99
				** **			Zpt	81.0±4.5	49.4±4.8	<0.001	**<0.001**
Muscle duty factor (% recovery phase)	Behav	118.43	1346	**<0.001**	0.44	0.45	Abd	86.2±3.6	39.7±3.2	<0.001	**<0.001**
	Muscle	48.43	3346	**<0.001**			Add	34.4±3.5	82.4±3.3	<0.001	**<0.001**
	Behav×Muscle	112.45	3346	**<0.001**			Cmt	72.9±3.6	49.4±3.2	<0.001	**<0.001**
				** **			Zpt	60.1±3.6	94.3±3.2	<0.001	**<0.001**
Full-cycle muscle RIA (% maximum)	Behav	18.83	1343	**<0.001**	0.31	0.32	Abd	5.19±0.61	9.32±0.77	<0.001	**<0.001**
	Muscle	5.86	3343	**<0.001**			Add	7.25±0.59	8.00±0.77	0.43	>0.99
	Behav×Muscle	13.52	3343	**<0.001**			Cmt	4.16±0.59	12.51±0.77	<0.001	**<0.001**
				** **			Zpt	4.57±0.61	11.99±0.77	<0.001	**<0.001**
Propulsive-phase muscle RIA (% maximum)	Behav	35.68	1343	**<0.001**	0.31	0.40	Abd	2.09±0.64	5.43±0.58	<0.001	**<0.001**
	Muscle	14.36	3343	**<0.001**			Add	5.41±0.62	5.87±0.65	0.44	>0.99
	Behav×Muscle	5.89	3343	**<0.001**			Cmt	2.80±0.57	6.46±0.74	<0.001	**<0.001**
				** **			Zpt	4.41±0.58	7.77±0.96	<0.001	**0.004**
Recovery-phase muscle RIA (% maximum)	Behav	0.09	1346	0.76	0.29	0.30	Abd	4.92±0.58	4.65±0.69	0.76	>0.99
	Muscle	6.46	3346	**<0.001**			Add	2.72±0.32	4.60±0.38	<0.001	**0.001**
	Behav×Muscle	10.68	3346	**<0.001**			Cmt	2.16±0.35	5.96±0.42	<0.001	**<0.001**
				** **			Zpt	3.33±0.40	8.23±0.47	<0.001	**<0.001**
Full-cycle EMG maximum amplitude (% maximum)	Behav	20.87	1343	**<0.001**	0.32	0.42	Abd	30.7±5.2	56.6±5.9	<0.001	**<0.001**
	Muscle	2.75	3343	**0.043**			Add	39.0±4.8	47.8±5.5	0.067	>0.99
	Behav×Muscle	7.63	3343	**<0.001**			Cmt	27.3±5.0	65.5±5.7	<0.001	**<0.001**
				** **			Zpt	30.9±4.7	66.5±5.1	<0.001	**<0.001**
Propulsive-phase EMG maximum amplitude (% maximum)	Behav	75.34	1343	**<0.001**	0.34	0.42	Abd	8.6±4.6	54.0±5.7	<0.001	**<0.001**
	Muscle	19.80	3343	**<0.001**			Add	36.7±4.4	36.7±5.7	0.96	>0.99
	Behav×Muscle	20.70	3343	**<0.001**			Cmt	22.0±4.5	57.8±5.7	<0.001	**<0.001**
				** **			Zpt	28.7±4.5	30.5±5.7	0.73	>0.99
Recovery-phase EMG maximum amplitude (% maximum)	Behav	0.79	1346	0.38	0.32	0.39	Abd	30.4±4.8	34.5±4.9	0.38	>0.99
	Muscle	5.57	3346	**0.001**			Add	14.9±4.6	36.5±4.8	<0.001	**<0.001**
	Behav×Muscle	13.01	3346	**<0.001**			Cmt	17.1±4.0	46.0±6.3	<0.001	**<0.001**
				** **			Zpt	21.0±4.6	65.3±5.1	<0.001	**<0.001**
Number of EMG bursts (per full cycle)	Behav	111.26	1346	**<0.001**	0.39	0.47	Abd Add	1.61±0.12 1.88±0.12	2.53±0.14 2.80±0.14	<0.001	**<0.001**
										<0.001	**<0.001**
	Muscle	7.54	3346	**<0.001**			Cmt	1.70±0.12	2.62±0.14	<0.001	**<0.001**
				** **			Zpt	1.44±0.13	2.36±0.14	<0.001	**<0.001**
Number of EMG bursts (per propulsive phase)	Behav	0.09	1343	0.76	0.55	0.55	Abd	1.28±0.05	1.31±0.11	0.76	>0.99
	Muscle	6.32	3343	**<0.001**			Add	1.02±0.04	2.00±0.11	<0.001	**<0.001**
	Behav×Muscle	16.21	3343	**<0.001**			Cmt	1.07±0.04	1.19±0.11	0.31	>0.99
				** **			Zpt	1.10±0.04	1.94±0.11	<0.001	**<0.001**
Number of EMG bursts (per recovery phase)	Behav	10.38	1346	**0.001**	0.17	0.19	Abd	1.07±0.07	1.47±0.12	0.001	**0.022**
	Muscle	4.42	3346	**0.004**			Add	1.28±0.07	1.51±0.12	0.064	>0.99
	Behav×Muscle	7.54	3346	**<0.001**			Cmt	1.17±0.07	1.10±0.11	<0.001	**<0.001**
				** **			Zpt	1.35±0.07	1.11±0.11	0.051	0.81

ANOVA-type results of linear mixed-effects models testing differences in muscle activity between swimming and walking. Statistically significant *P*-values are in bold. *P*-values on the left of the table are for the ANOVA-type results. *P*-values on the right of the table are for pairwise comparisons between the estimated marginal means of swimming and walking. Abd, abductor; Add, adductor; Behav, behaviour; Cmt, coracometapterygialis; EMG, electromyography; emmean, estimated marginal mean; Muscle, pectoral fin muscle; *P*_bonf_, Bonferroni-corrected *P*-value; RIA, rectified integrated area; *R*^2^_m_, marginal r-squared (fixed effects only); *R*^2^_c_, conditional r-squared (whole model); Zpt, zonopropterygialis.

**Fig. 5.**
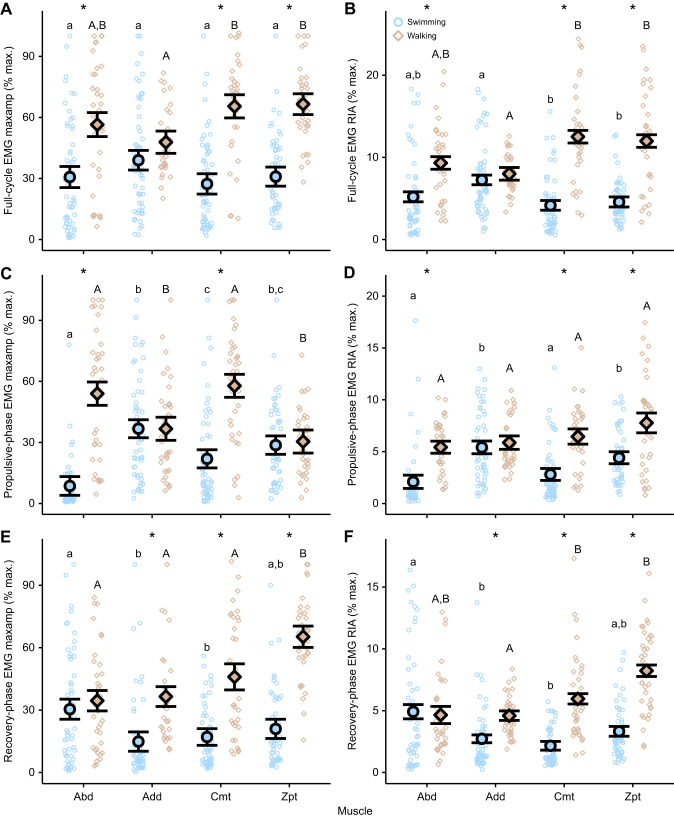
**Pectoral fin muscle activity magnitude differences between swimming and walking *P. senegalus*.** Muscle activity was recorded from four muscle groups in the pectoral fin during routine swimming and walking in *P. senegalus* (*N*=4). (A,B) Represent EMG maximum amplitude and EMG RIA (rectified integrated area) for the full stroke cycle. (C,D) Represent EMG maximum amplitude and EMG RIA for the propulsive-phase only. (E,F) Represent EMG maximum amplitude and EMG RIA for the recovery-phase only. Small circles are values from each cycle or phase. Larger points and error bars are the estimated marginal mean±s.e.m. for each behaviour. Letters denote means that are statistically different within a behaviour (Bonferroni-corrected *post hoc* comparisons; *P*<0.05; lower-case letters are differences during swimming, upper-case letters are differences during walking). Asterisks denote a significant difference between behaviours within a muscle (Bonferroni-corrected *post hoc* comparisons; *P*<0.05). Abd, abductor; Add, adductor; Cmt, coracometapterygialis; EMG, electromyography; max., maximum; maxamp, maximum amplitude; RIA, rectified integrated area; Zpt, zonopropterygialis.

Over the full cycle, EMG RIA and EMG maximum amplitude were higher during walking than during swimming in all muscles but the Add (Fig. 5A,B, Table 3). Specifically, EMG RIA was higher during walking than during swimming in the Abd, Cmt and Zpt muscles during propulsion (Fig. 5D, Table 3) and for the Add, Cmt and Zpt during recovery (Fig. 5F, Table 3). EMG maximum amplitude also showed large increases during walking compared with during swimming in the Abd and the Cmt (Fig. 5C, Table 3). During the recovery stroke, EMG maximum amplitude of Add, Cmt and Zpt increased during walking compared with during swimming (Fig. 5E, Table 3).

**Fig. 6.**
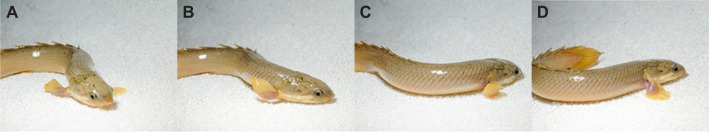
**Right pectoral fin positions during walking in *P. senegalus*.** (A–D) Right pectoral fin positions are shown at the beginning of the recovery phase (A), in the middle of the recovery phase (B), at the beginning of the propulsive phase (C) and in the middle of the propulsive phase (D). Images courtesy of Antoine Morin.

### Differences between muscles for a given locomotor mode

Within each locomotor mode, EMG duty factor and muscle activation duration differed between muscles (Fig. 4, Table 3). During walking and calculated over the full stroke cycle, muscle activation duration and EMG duty factor of the Add and Zpt were significantly higher than for the Abd and Cmt (Fig. 4A,B, Tables 3 and 4). These differences were accounted for because, during walking, the Add and Zpt muscles remained active for longer during the recovery stroke compared with the Abd and Cmt (Fig. 4E,F, Tables 3 and 4). In contrast, over the full stroke cycle during swimming, EMG duty factor and muscle activation duration did not differ for Abd and Cmt; however, the Zpt had higher muscle activation duration and EMG duty factor than the Add (Fig. 4A,B, Tables 3 and 4). Although there were few differences across the entire cycle during swimming, there were differences in EMG duration and EMG duty factor within the propulsive and recovery portion of the stroke (Fig. 4C–F). During the propulsive phase, the Add and Zpt had longer duration and greater duty factor than the Abd and Cmt (Fig. 4C,D). In contrast, during recovery, Add and Zpt tended to have shorter duration and smaller duty factors than the Abd and Cmt (Fig. 4E,F), explaining the lack of statistical difference between muscles across the entire stroke cycle (Tables 3 and 4).

**Table 4.** Pairwise comparisons of muscle activity between muscles during swimming and walking in *P. senegalus*

Variable	Muscle	Swimming		Walking	
		*P*	*P*_bonf_	*P*	*P*_bonf_
Full-cycle muscle activation duration (s)	Abd vs Add	0.044	0.71	<0.001	**<0.001**
	Abd vs Cmt	0.82	>0.99	0.71	>0.99
	Abd vs Zpt	0.20	>0.99	<0.001	**<0.001**
	Add vs Cmt	0.023	0.37	<0.001	**<0.001**
	Add vs Zpt	0.001	**0.016**	0.80	>0.99
	Cmt vs Zpt	0.28	>0.99	<0.001	**<0.001**
Propulsive-phase muscle activation duration (s)	Abd vs Add	<0.001	**<0.001**	0.42	>0.99
	Abd vs Cmt	0.004	0.061	0.037	0.59
	Abd vs Zpt	<0.001	**<0.001**	0.001	**0.017**
	Add vs Cmt	<0.001	**<0.001**	0.20	>0.99
	Add vs Zpt	0.73	>0.99	0.013	0.20
	Cmt vs Zpt	<0.001	**0.011**	0.23	>0.99
Recovery-phase muscle activation duration (s)	Abd vs Add	<0.001	**<0.001**	<0.001	**<0.001**
	Abd vs Cmt	0.007	0.11	0.009	0.14
	Abd vs Zpt	<0.001	**<0.001**	<0.001	**<0.001**
	Add vs Cmt	<0.001	**<0.001**	<0.001	**<0.001**
	Add vs Zpt	<0.001	**<0.001**	0.005	0.082
	Cmt vs Zpt	0.019	0.30	<0.001	**<0.001**
Muscle duty factor (% full cycle)	Abd vs Add	0.045	0.72	>0.001	**<0.001**
	Abd vs Cmt	0.87	>0.99	0.80	>0.99
	Abd vs Zpt	0.16	>0.99	<0.001	**<0.001**
	Add vs Cmt	0.028	0.45	<0.001	**<0.001**
	Add vs Zpt	<0.001	**0.010**	0.65	>0.99
	Cmt vs Zpt	0.20	>0.99	<0.001	**<0.001**
Muscle duty factor (% propulsive phase)	Abd vs Add	<0.001	**<0.001**	0.41	**>0.99**
	Abd vs Cmt	0.002	**0.026**	0.002	**0.027**
	Abd vs Zpt	<0.001	**<0.001**	<0.001	**0.001**
	Add vs Cmt	<0.001	**<0.001**	0.042	0.67
	Add vs Zpt	0.64	>0.99	0.002	**0.034**
	Cmt vs Zpt	<0.001	**0.002**	0.11	**>0.99**
Muscle duty factor (% recovery phase)	Abd vs Add	<0.001	**<0.001**	<0.001	**<0.001**
	Abd vs Cmt	0.003	0.055	0.015	0.24
	Abd vs Zpt	<0.001	**<0.001**	<0.001	**<0.001**
	Add vs Cmt	<0.001	**<0.001**	<0.001	**<0.001**
	Add vs Zpt	<0.001	**<0.001**	0.003	0.052
	Cmt vs Zpt	0.005	0.081	<0.001	**<0.001**
Full-cycle muscle RIA (% maximum)	Abd vs Add	0.01	0.19	0.21	>0.99
	Abd vs Cmt	0.20	>0.99	0.003	**0.04**
	Abd vs Zpt	0.45	>0.99	0.01	0.19
	Add vs Cmt	<0.001	**0.002**	<0.001	**<0.001**
	Add vs Zpt	0.001	**0.02**	0.0002	**0.003**
	Cmt vs Zpt	0.61	>0.99	0.62	>0.99
Propulsive-phase muscle RIA (% maximum)	Abd vs Add	<0.001	**<0.001**	0.43	>0.99
	Abd vs Cmt	0.20	>0.99	0.12	>0.99
	Abd vs Zpt	<0.001	**<0.001**	0.010	0.16
	Add vs Cmt	<0.001	**<0.001**	0.42	>0.99
	Add vs Zpt	0.060	0.95	0.046	0.73
	Cmt vs Zpt	<0.001	**0.013**	0.19	>0.99
Recovery-phase muscle RIA (% maximum)	Abd vs Add	<0.001	**0.010**	0.94	>0.99
	Abd vs Cmt	<0.001	**<0.001**	0.099	>0.99
	Abd vs Zpt	0.020	0.32	<0.001	**<0.001**
	Add vs Cmt	0.20	>0.99	0.012	0.19
	Add vs Zpt	0.21	>0.99	<0.001	**<0.001**
	Cmt vs Zpt	0.022	0.35	<0.001	**0.003**
Full-cycle EMG maximum amplitude (% maximum)	Abd vs Add	0.069	>0.99	0.14	>0.99
	Abd vs Cmt	0.47	>0.99	0.14	>0.99
	Abd vs Zpt	0.96	>0.99	0.072	>0.99
	Add vs Cmt	0.007	0.11	0.002	**0.032**
	Add vs Zpt	0.041	0.65	<0.001	**0.005**
	Cmt vs Zpt	0.39	>0.99	0.84	>0.99
Propulsive-phase EMG maximum amplitude (% maximum)	Abd vs Add	<0.001	**<0.001**	0.005	0.089
	Abd vs Cmt	<0.001	**0.008**	0.53	>0.99
	Abd vs Zpt	<0.001	**<0.001**	<0.001	**0.002**
	Add vs Cmt	<0.001	**<0.001**	<0.001	**0.011**
	Add vs Zpt	0.029	0.47	0.31	>0.99
	Cmt vs Zpt	0.071	>0.99	<0.001	**<0.001**
Recovery-phase EMG maximum amplitude (% maximum)	Abd vs Add	<0.001	**0.005**	0.66	>0.99
	Abd vs Cmt	<0.001	**0.004**	0.061	0.98
	Abd vs Zpt	0.029	0.46	<0.001	**<0.001**
	Add vs Cmt	0.51	>0.99	0.11	>0.99
	Add vs Zpt	0.14	>0.99	<0.001	**<0.001**
	Cmt vs Zpt	0.25	>0.99	0.002	**0.036**
Number of EMG bursts (per full cycle)	Abd vs Add	0.004	**0.025**	0.004	**0.025**
	Abd vs Cmt	0.33	>0.99	0.33	>0.99
	Abd vs Zpt	0.080	0.48	0.080	0.48
	Add vs Cmt	0.056	0.34	0.056	0.34
	Add vs Zpt	<0.001	**<0.001**	<0.001	**<0.001**
	Cmt vs Zpt	0.006	**0.037**	0.006	**0.037**
Number of EMG bursts (per propulsive phase)	Abd vs Add	<0.001	**<0.001**	<0.001	**<0.001**
	Abd vs Cmt	0.001	**0.020**	0.45	>0.99
	Abd vs Zpt	0.005	0.087	<0.001	**0.002**
	Add vs Cmt	0.35	>0.99	<0.001	**<0.001**
	Add vs Zpt	0.18	>0.99	0.73	>0.99
	Cmt vs Zpt	0.68	>0.99	<0.001	**<0.001**
Number of EMG bursts (per recovery phase)	Abd vs Add	0.012	0.19	0.80	>0.99
	Abd vs Cmt	0.24	>0.99	0.14	>0.99
	Abd vs Zpt	<0.001	**0.013**	0.019	0.30
	Add vs Cmt	0.18	>0.99	0.22	>0.99
	Add vs Zpt	0.38	>0.99	0.010	0.16
	Cmt vs Zpt	0.028	0.45	<0.001	**0.002**

Statistically significant Bonferroni-corrected *P*-values are shown in bold. Note that because the model for number of EMG bursts (per cycle) is a non-interaction model, the *P*-values for swimming and walking are the same. Abd, abductor; Add, adductor; Cmt, coracometapterygialis; EMG, electromyography; RIA, rectified integrated area; Zpt, zonopropterygialis.

Muscle effort (RIA) and maximum EMG amplitude differed between muscles for both modes of locomotion (Table 1). During walking, EMG RIA and EMG maximum amplitudes over the full cycle were significantly lower in the Add than in the Cmt and Zpt (Fig. 5A,B, Tables 3 and 4). These differences were primarily due to a lower Add EMG RIA during the recovery phase of the stroke (Fig. 5F) and higher EMG maximum amplitude during propulsion in the Cmt (Fig. 5C) and during recovery in the Zpt (Fig. 5E, Tables 3 and 4).

During swimming, over the full cycle, EMG maximum amplitude did not differ between muscles (Fig. 5A, Tables 3 and 4); however, EMG maximum amplitude did differ by stroke phase for the Abd muscle as it was lower during the propulsive phase and higher during the recovery phase than for most other muscles (Fig. 5C,E, Tables 3 and 4). Full-cycle EMG RIA during swimming differed between muscles, with the Add having a higher value than the Cmt and Zpt (Fig. 5B, Tables 3 and 4). These differences appeared to be driven primarily by an increase in Add EMG RIA during the propulsive phase of the stroke (Fig. 5D, Table 4). The Abd muscle also showed lower EMG RIA activity during propulsion and higher EMG RIA activity during recovery (Fig. 5D,F, Table 4).

### Changes in pattern of activation

All muscles had more bursts per cycle during walking than during swimming (Table 3, Fig. S1A); the Add and Zpt had more bursts during the propulsive phase, and the Abd and Cmt had more bursts during the recovery phase (Table 3, Fig. S1B,C).

Comparing the muscles, during swimming, the Add and Cmt had significantly fewer bursts than the Abd during propulsion (Tables 3 and 4, Fig. S1B), whereas during walking the Add and Zpt had significantly more bursts than the Abd and Cmt during propulsion (Table 4, Fig. S1B). During the swimming recovery phase, the Abd had fewer bursts, and the Zpt had more bursts, than the other muscles (Tables 3 and 4, Fig. S1C). During the walking recovery phase, the Cmt tended to have more bursts, and the Zpt had fewer bursts, than the other muscles (Tables 3 and 4, Fig. S1C).

Muscle activity onset and offset displayed consistent timing during both swimming and walking for all muscles except for the Add (Fig. 3, Table S1). Likewise, muscle activity onset and offset timing was significantly different between swimming and walking for all muscles where it could be evaluated (excluding the Add, owing to inconsistent timing during walking; Fig. 3, Table 2).

### Cell damage following terrestrial walking

The patterns of cellular membrane permeability (a proxy for muscle damage) in pectoral fin muscles varied across fish of different sizes (Fig. S2, Table S2). Whereas the largest fish we investigated showed no significant changes in muscle damage following walking, all pectoral fin muscles of small and medium fish that experienced exhaustive terrestrial walking had more intense Evans Blue dye staining than in those of non-walked fish (Fig. S2, Table S2). Moreover, the effect sizes for differences are all non-zero, suggesting that the magnitude of the difference is biologically relevant (Fig. S2, Table S2). Staining of cells had a distributed speckling pattern, as can be seen in Fig. S3. In addition, although not significant, staining in the Abd, Add and Zpt tended to be greater at more distal than proximal positions on the fin (Fig. S2, Table S2). Staining in the Cmt had no proximal–distal gradient (Fig. S2, Table S2). Also, differences in the proximal–distal gradient of the Add muscle in small fish had a significant interaction term (Fig. S2, Table S2).

## DISCUSSION

Fish must overcome extra force due to gravity on land (Ashley-Ross et al., 2013; Wright and Turko, 2016). They must also change the position of their body and fins during motion to produce the appropriate forces in each environment. These positional changes can change the range of muscle operating length, required contraction speed, required force and whether muscles are active while lengthening or shortening, all of which affect muscle function. Below we provide a descriptive analysis of muscle activation during swimming and walking and then discuss how differences in muscle functional performance generate hypotheses of mechanisms driving fin muscle plasticity elicited by terrestrial environments.

### Muscle activation patterns during swimming and walking differ

During swimming, fin muscles work together to move the fin through a smooth oscillation of adduction and abduction (Fig. 3A). As expected, the Add and Abd muscles work in sequence to actively move the fin towards and away from the body, respectively (Foster et al., 2018; Lutek et al., 2022b; Fig. 3BC). At the same time, the Zpt and Cmt work by co-contracting with either the Add or Abd to expand and feather the fin surface appropriately (Fig. 3D,E). As the Add begins to pull the fin towards the body in the swimming propulsive stroke, the Cmt keeps the lower edge of the fin expanded downward, helping to maximize the fin area pulling water. As the propulsive stroke continues and the fin gets closer to the body, the Cmt turns off and the Zpt becomes active to help expand the fin's upper edge. During fin stroke recovery, when the Add is no longer active, the Cmt starts the fin abduction by feathering the ventral edge of the fin out, reducing drag as the Abd activates and pulls the fin forward. The swimming stroke has relatively steady and slow fin motion, and muscle contraction is dominated by concentric or isometric contractions (Fig. 1D, Fig. 3B–E). Nose elevation does not change throughout the stroke, and fin elevation rises and falls with the onset and offset of the Zpt and Cmt, as expected (Fig. 3A,D,E).

During walking, muscle activation timing changes relative to fin position, altering muscle functional performance (Fig. 3G–J, Fig. 4). Most notably, the Add muscle is active for the majority of the stroke cycle, co-contracting with the Abd and the Cmt during stance and with the Zpt during swing. During stance, the antagonistic action of the Add and Abd, combined with the depressor function of the Cmt, work together to keep the fin firmly placed on the ground with the lateral side of the fin facing down. The start of stance is a forceful part of the stroke; as the three muscles contract, the body is lifted over the fin and reaches maximum nose elevation and maximum fin adduction early in the stance cycle (Fig. 3F). Quickly, the nose falls, in a rather ballistic trajectory, reaching its lowest elevation (see fig. 1 in Standen et al., 2014). The Add has a brief moment of inactivity as the Zpt starts to lift the fin in preparation for the swing cycle, and, because the medial side of the fin is facing up at this point in the stroke, the Add co-activates with the Zpt, which curves the fin upward and pulls it forward. This can appear as a sweeping motion and results in a dip and rise in the fin elevation throughout swing (see fig. 1 in Standen et al., 2014). The walking stroke has both rapid and slower periods of motion, and the Add muscle has a clear moment of strong eccentric contraction while nose elevation increases to raise the fish up over the fin during stance.

### Eccentric contraction and co-contraction

The Add muscle appears to experience the largest difference in functional performance between swimming and walking (activation throughout the cycle; Fig. 1E, Fig. 3H). At the beginning of stance during walking, there is a burst of co-contraction of the Add, Abd and Cmt (Fig. 1E, Fig. 3G–I). During this period of loading, the Add and, possibly, the Cmt are stretched longer than their regular swimming range (Fig. 2G; smaller angles here mean greater abduction of the fin and stretching of the Add and possibly Cmt). In addition, the Add is stretched as it contracts during the first part of stance while the fin continues to abduct, and the head and body are loaded onto, and lifted over, the fin (Fig. 3B,H). Both eccentric contraction and activating at extended muscle lengths may lead to muscle damage (Child et al., 1998; Morgan and Allen, 1999; Proske and Morgan, 2001). Preliminary data we collected on muscle permeability post walking show greater muscle cell permeability (damage) in all fin muscles of medium and small fish (Fig. S2, Table S2). Antagonistic contraction between the Add and Abd may also contribute to some muscle fibre damage by holding the fin in a posture that, when loaded with the rather ballistic heave of the body over the fin, results in considerable muscle loading. As the muscles pull against each other under larger load, brief periods of imbalance could lead to momentary eccentric bouts that produce micro-tears in muscle fibres (Child et al., 1998). Both the Zpt and Cmt regularly co-contract with the Add and Abd muscles during swimming. While walking, the change in contraction velocity and force environments (loading) during co-contraction may be what causes damage in these smaller muscle groups (Fig. 2K,L). In addition, range of motion significantly increases in both the horizontal (fin adduction angle) and vertical (fin elevation) plane during walking (Fig. 2D,H), suggesting that, to some degree, all pectoral fin muscles are stretched to longer lengths during walking than during swimming. These ‘deviations’ from the normal range of motion could disrupt sarcomere alignment and cause muscle damage, as is seen in many sports physiology studies (Baroni et al., 2017; Jones et al., 1989; Newham et al., 1988; Nosaka and Sakamoto, 2001).

*Polypterus senegalus* that are raised on land show a shift in muscle fibre type to glycolytic muscle (Du and Standen, 2017). This is somewhat counter intuitive as the consensus in the literature is that type II fibres are more likely to experience (or are at least more susceptible to) damage for a variety of structural reasons (e.g. Macaluso et al., 2012; McKune et al., 2012; Qaisar et al., 2016). However, some evidence suggests that ballistic/plyometric exercise can induce shifts away from aerobic type 1 fibres (towards type II fibres), possibly explaining what is seen in the fish (reviewed in Plotkin et al., 2021). Some might also argue that microdamage signals muscle remodelling, not necessarily by changing fibre type, but rather by upregulating protein synthesis to increase muscle cross-sectional area and thus increase force capacity (Qaisar et al., 2016). Although this is a possibility, *P. senegalus* fibre-type changes are not accompanied by muscle hypertrophy; in fact, muscles were reported to be smaller in exercised fish than in size-matched controls (Du and Standen, 2017).

### Contraction velocity and impulse of loading

Previous studies have shown an increase in the white or anaerobic muscle fibres in fin muscles of fish that were terrestrialized for 2 months (Du and Standen, 2017). Looking at muscle activation patterns in walking compared with swimming (Fig. 5), we see that overall muscle effort, as defined by RIA (Fig. 5B,D,F), is significantly higher during walking than during swimming. This increase in muscle effort is due to increases in muscle activation amplitude (Fig. 5A,C,E) and muscle activation duration (Fig. 4A,C,E), suggesting that both the intensity and duration of contraction are contributing to muscle remodelling in this system. Although fin muscles were too small to measure the shortening rate directly, maximum and routine fin adduction velocities were higher during walking than during swimming (Fig. 2K,L), suggesting that muscle contraction rate was also increased during walking. Finally, although we did not measure force magnitudes in this study, higher amplitude muscle activation, higher speed fin movement and the fast transition from fin plant to maximum head elevation suggest that the impulse associated with loading fins during the first part of the step cycle is higher during walking than during swimming and may be a contributing factor to the muscle membrane permeability and resultant fibre change that we see in these fish.

### Prolonged muscle use and fatigue

Prolonged use of a muscle without a chance to recover also leads to muscle fatigue and damage (Appell et al., 1992; Coso et al., 2012; Hunter et al., 2012). Add and Zpt muscles turned on for a longer absolute time during walking than during swimming (Fig. 4A). Interestingly, the Add was used for the longest duration during walking compared with other fin muscles (Fig. 4AB). Although Foster et al. (2018) suggested that the Add produced double bursts within a stroke when walking, our data show the Add to have trains of bursts in a single cycle that result in relatively sustained contraction during both stance and swing, remaining active for close to the entire fin beat cycle (Fig. 1, Fig. 3C,H, Fig. 4B,F). Consequently, there was little time for the Add to recover between fin steps, which could ultimately fatigue the muscle and influence physiological processes that lead to muscle fibre change. All muscles in the fin experienced an increase in the number of bursts per cycle (Fig. S1), suggesting that changing locomotor mode from swimming to walking has a significant functional impact on the physiology of the muscle.

### Measuring muscle cell permeability

Muscle activity levels in combination with fin movements suggest that muscles are operating outside of their regular range of motion and at an increased effort during walking. To gain more insight into the physiological impact of this biomechanical change, we used Evans Blue dye to measure membrane permeability in muscles post walking. Although this technique is often used in conjunction with immunohistochemistry, we used it here as a proxy for muscle damage (Hamer et al., 2002). In animals that walked, we saw an increase in muscle cell permeability, suggesting that the change in muscle use causes small-scale muscle damage in small and medium fish (Fig. S2, Table S2). Scaling laws would suggest that large fish should be even more susceptible to muscle damage; however, we do not see a statistical increase in cell permeability in this size of fish. This may be due to the smaller sample size or to geometric changes over ontogeny that alter mechanical loading from muscle to bone. Small-scale muscle tearing is a molecular mechanism that leads to muscle fibre-type shifts (Matsuura et al., 2007). The fact that some muscle damage is seen in our fish suggests that walking contributes to muscle damage, which could lead to the distinct muscle fibre changes in terrestrialized *P. senegalus* (Du and Standen, 2017; length±s.e.m., 70±15 mm; weight±s.e.m., 2.85±2.65 g).

### Conclusion: environment elicits paradoxical muscle functional change

Longer-term behavioural changes in animals, whether aquatic or terrestrial, are often accompanied by changes in muscle fibre size and type (Brunt et al., 2016; Sänger, 1993). *Polypterus senegalus* muscle fibres change from red to white after being raised on land for extended periods of time (Du and Standen, 2017). The present study looked at instantaneous changes in fin use (behavioural plasticity) that *P. senegalus* exhibit when exposed to a terrestrial environment and the resultant novel function imposed on muscles, to help explain the specific drivers of the plastic response seen in tissues after terrestrial acclimation. In this study, walking behaviour differs from swimming behaviour; we see increases in the operating length of muscles, eccentric and antagonistic co-contraction, rate of force production and velocity of contraction, as well as prolonged muscle use. These data show the remarkable flexibility of a group of pectoral muscles that, by making quite small changes in the timing and magnitude of activation, can dramatically alter overall fin function. These observations generate the hypothesis that muscle fibre remodelling can be elicited by quite subtle differences in muscle use and warrant future study using an inverse dynamics approach to measure true forces and individual muscle geometry to clarify the largest contributors to performance change between walking and swimming locomotor modes.

## Supplementary Material

10.1242/jexbio.250474_sup1 Supplementary information

Dataset 1. Evan's Blue Dye data.

Dataset 2. Electromyography data.

Dataset 3. Kinematic data.

Dataset 4. This is the R script used to analyze the data in this study.

Dataset 5. This is the Rproject used to produce the statistical analysis in this study.

Dataset 6. Code instructions.
