# Supplementary material for: Walking elicits muscle functional changes in the pectoral fin of Polypterus senegalus
Source: J Exp Biol. 2025 Nov 6;228(21):jeb250474. doi: 10.1242/jeb.250474 (PMC12633731; doi:10.1242/jeb.250474)
Supplement: Supplementary information [file jexbio-228-250474-s1.pdf]

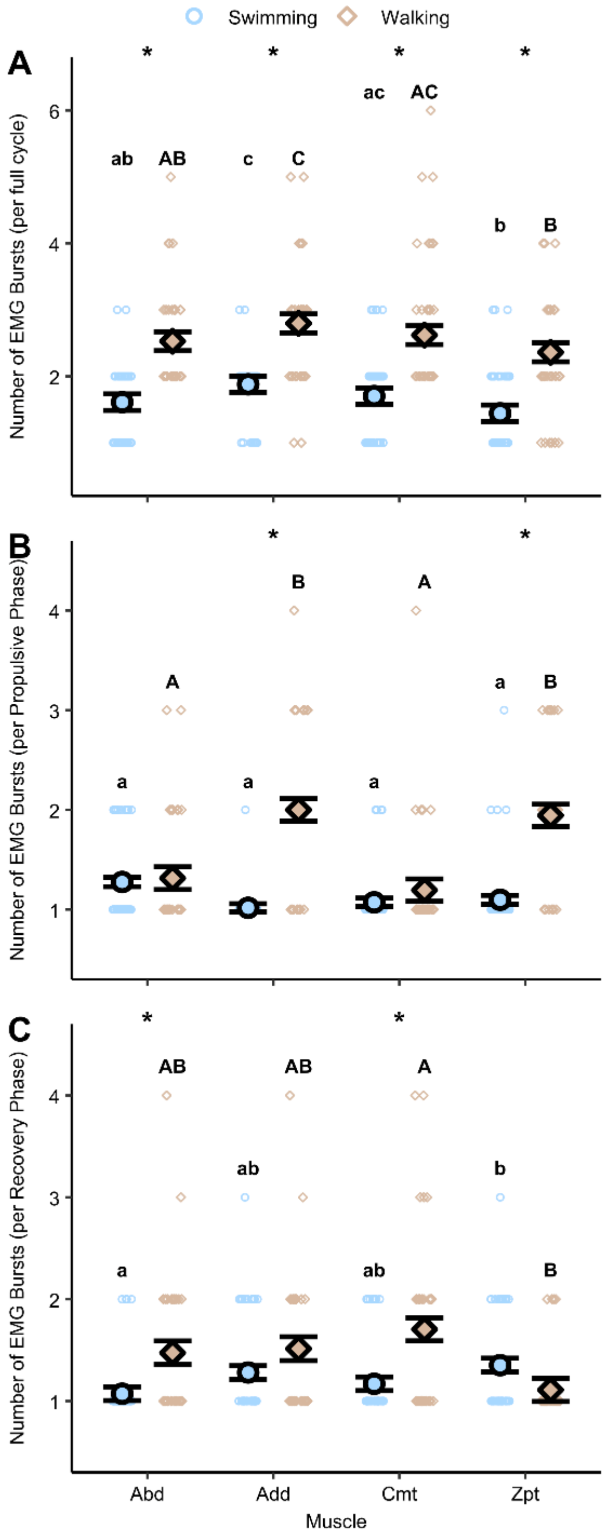

Journal of Experimental Biology • Supplementary information

Fig. S1. Pectoral fin muscle activity burst number differences between swimming and walking *Polypterus senegalus* (n=4). Muscle activity was recorded from four muscle groups in the pectoral fin during routine swimming and walking in *P. senegalus*. Small circles are values from each cycle or phase. Large points and error bars are the estimated marginal mean ± s.e.m. for each behaviour. Letters denote means that are statistically different within a behaviour (Bonferroni-corrected post-hoc comparisons; $p < 0.05$; lower case letters are differences during swimming, upper case letters are differences during walking). Asterisks denote a significant difference between behaviours within a muscle (Bonferroni-corrected post-hoc comparions; $p < 0.05$). Note that for the number of EMG bursts per cycle the best model fit is a non-interaction model, but statistical results are presented as for other variables for consistency. Abd, abductor; Add, adductor; Cmt, coracometapterygialis; EMG, electromyography; MaxAmp, maximum amplitude; RIA, rectified integrated area; Zpt, zonopropterygialis.

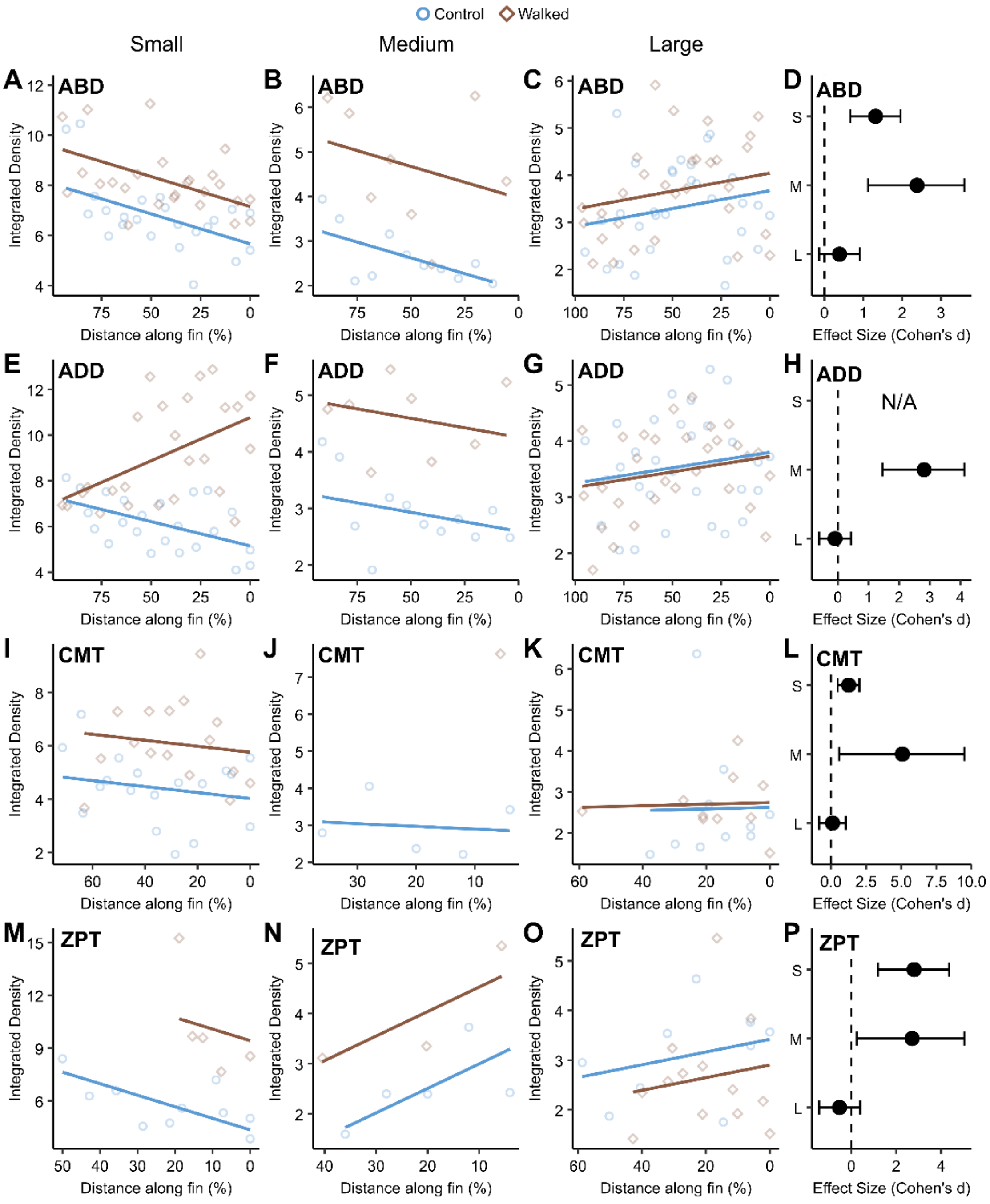

Journal of Experimental Biology • Supplementary information

Fig. S2. Muscle damage increases following walking in small (n=4) and medium (n=2) fish, but not large fish (n=6). Muscle damage was assessed for fish that had not walked (red; Control) and fish that had been walked to exhaustion (blue; Exercise). Distance along the fin goes from proximal (0%) to distal (100%). Points in columns 1-3 are measurements from individual slices and lines show a linear regression best fit. The points and error bars in column 4 are estimates of Cohen's d and a 95% confidence interval for the effect of treatment on integrated density based on t test statistics from the linear regressions. We could not determine an appropriate effect size estimate for the abductor of small fish as there is a significant interaction in the model. Small and medium exercised fish had significantly more muscle damage than their controls, while large fish did not experience muscle damage (linear regression). Abd, abductor; Add, adductor; Cmt, coracometapterygialis; Zpt, zonopropterygialis.

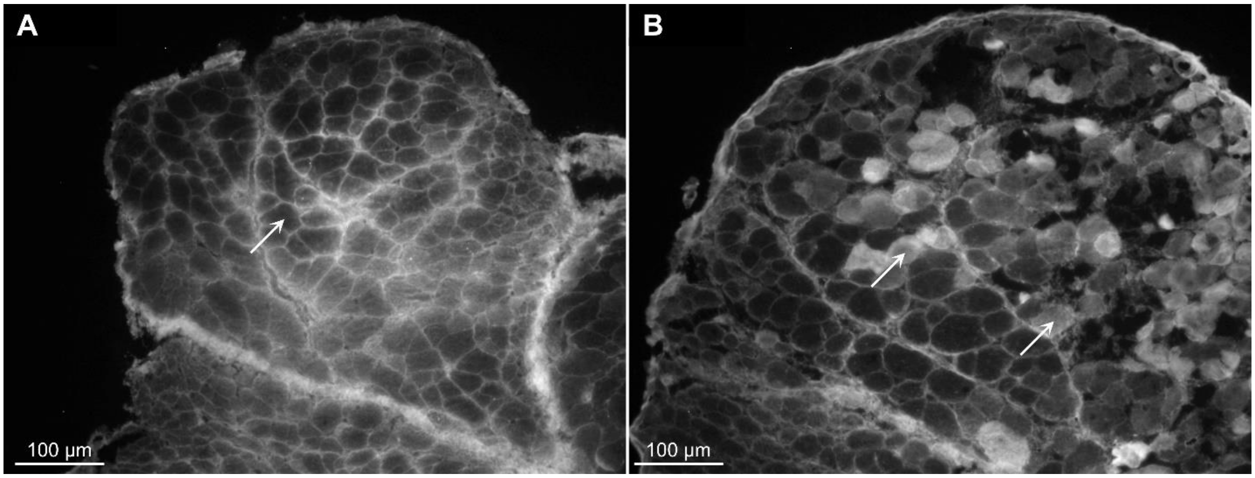

Fig. S3. Sections of the coracometapterygialis from the right pectoral fin from (A) a fish that had not walked and (B) a fish that had been walked to exhaustion. Note that Evans Blue Dye (EBD) is visible in the interstitial spaces between cells. Arrows indicate individual cells that exemplify the differences in EBD uptake between fish from the two treatments. This uptake is a result of myofibers being damaged or permeable.

Table S1. Circular distributions of kinematic and muscle activity variables during swimming and walking in *Polypterus senegalus*.

		Swim				Walk			
		von Mises			Uniformity	von Mises			Uniformity
Variable	Muscle	κ	K	p	p	κ	K	p	p
Maximum pectoral fin elevation	-	1.54	504	>0.99	**<0.0001**	0.96	224	>0.99	**0.0045**
Minimum pectoral fin elevation	-	1.37	294	>0.99	**<0.0001**	0.59	224	>0.99	0.11
Maximum pectoral fin adduction angle	-	0.97	352	>0.99	**0.0002**	3.14	261	>0.99	**<0.0001**
Minimum pectoral fin adduction angle	-	1.10	462	>0.99	**<0.0001**	2.55	252	>0.99	**<0.0001**
Maximum nose elevation	-	-	-	-	**-**	4.98	196	>0.99	**<0.0001**
Minimum nose elevation	-	-	-	-	**-**	3.93	224	>0.99	**<0.0001**
Muscle activity onset	Abd	1.58	260	>0.99	**<0.0001**	1.07	1159	0.05	**0.0001**
	Add	2.24	472	>0.99	**<0.0001**	0.33	1309	>0.99	0.13
	Cmt	0.68	715	>0.99	**0.003**	1.67	816	>0.99	**<0.0001**
	Zpt	0.67	572	>0.99	**0.005**	1.19	540	>0.99	**<0.0001**
Muscle activity offset	Abd	1.46	424	>0.99	**<0.0001**	2.58	576	>0.99	**0.0002**
	Add	2.39	434	>0.99	**<0.0001**	0.27	924	>0.99	0.24
	Cmt	0.71	742	>0.99	**0.002**	0.44	1311	0.1	**0.04**
	Zpt	1.15	364	>0.99	**<0.0001**	1.79	693	>0.99	**<0.0001**

A Kuiper test (K, test statistic) was used to assess whether each variable's distribution was significantly different than a von Mises distribution with a kappa parameter (κ) and angular mean of the sample (H_o: the sample distribution is a von Mises distribution). When the variable had a von Mises distribution, Rayleigh's test was used to determine whether the variable was uniformly distributed throughout the cycle, otherwise a Herman-Rasson test (†) was used. Significant p-values are in bold.

Journal of Experimental Biology • Supplementary information

Table S2. Muscle damage following walking in *Polypterus senegalus*.

Variable	Fixed effect	F	df	p	R^2	d[CI]
Integrated Density, Small, Abd	Dist	16.24	1,45	**0.0002**	0.43	1.32[0.67,1.96]
	Treat	19.53	1,45	**<0.0001**		
Integrated Density, Medium, Abd	Dist	3.55	1,17	0.08	0.63	2.38[1.12,3.60]
	Treat	24.11	1,17	**0.0001**		
Integrated Density, Large, Abd	Dist	2.81	1,57	0.10	0.08	0.39[-0.14,0.91]
	Treat	2.15	1,57	0.15		
Integrated Density, Small, Add	Dist	3.21	1,44	0.08	0.57	N/A
	Treat	43.74	1,44	**<0.0001**		
	Dist×Treat	13.32	1,44	**0.0007**		
Integrated Density, Medium, Add	Dist	1.69	1,17	0.21	0.68	2.81[1.45,4.12]
	Treat	33.57	1,17	**<0.0001**		
Integrated Density, Large, Add	Dist	2.10	1,57	0.15	0.04	-0.09[-0.61,0.43]
	Treat	0.08	1,57	0.73		
Integrated Density, Small, Cmt	Dist	0.87	1,31	0.36	0.29	1.26[0.48,2.02]
	Treat	12.29	1,31	**0.001**		
Integrated Density, Medium, Cmt	Dist	0.05	1,3	0.85	0.89	5.09[0.58,9.50]
	Treat	19.40	1,3	**0.02**		
Integrated Density, Large, Cmt	Dist	0.01	1,17	0.92	0.01	0.11[-0.85,1.06]
	Treat	0.05	1,17	0.83		
Integrated Density, Small, Zpt	Dist	4.01	1,12	0.07	0.66	2.80[1.19,4.35]
	Treat	23.50	1,12	**0.0003**		
Integrated Density, Medium, Zpt	Dist	6.27	1,5	0.05	0.74	2.71[0.25,5.03]
	Treat	9.16	1,5	**0.03**		
Integrated Density, Large, Zpt	Dist	0.91	1,19	0.35	0.09	-0.52[-1.43,0.40]
	Treat	1.30	1,19	0.27		

Abd, abductor; Add, adductor; CI, 95% confidence interval; Cmt, coracometapterygialis; d, Cohen's d; Dist, distance along fin; Treat, treatment; Zpt, zonopropterygialis.

Journal of Experimental Biology • Supplementary information

Dataset 1. Evan's Blue Dye data. This dataset includes the integrated density for all muscles divided by fish size and treatment. This dataset is called by the supplied Rcode.

Available for download at
https://journals.biologists.com/jeb/article-lookup/doi/10.1242/jeb.250474#supplementary-data

Dataset 2. Electromyography data. This dataset includes all muscle activation variables calculated from raw EMG signals. EMG duration, duty factor, maximum amplitude, RIA and number of bursts are included for the full stroke cycle, propulsive phase and recovery phase. This dataset is called by the supplied Rcode.

Available for download at
https://journals.biologists.com/jeb/article-lookup/doi/10.1242/jeb.250474#supplementary-data

Dataset 3. Kinematic data. This dataset includes all calculated kinematic variables for individual trials of individual fish swimming and walking. This dataset is called by the supplied Rcode.

Available for download at
https://journals.biologists.com/jeb/article-lookup/doi/10.1242/jeb.250474#supplementary-data

Dataset 4. This is the R script used to analyze the data in this study.

Available for download at

https://journals.biologists.com/jeb/article-lookup/doi/10.1242/jeb.250474#supplementary-data

Dataset 5. This is the Rproject used to produce the statistical analysis in this study.

Available for download at

https://journals.biologists.com/jeb/article-lookup/doi/10.1242/jeb.250474#supplementary-data

Dataset 6. Code instructions. This readme file includes detailed instructions on how to use the supplementary code and data files to recreate the analysis conducted in this study.

Available for download at
https://journals.biologists.com/jeb/article-lookup/doi/10.1242/jeb.250474#supplementary-data

Journal of Experimental Biology • Supplementary information
