# Supplementary material for: Walking elicits muscle functional changes in the pectoral fin of Polypterus senegalus
Source: J Exp Biol. 2025 Nov 6;228(21):jeb250474. doi: 10.1242/jeb.250474 (PMC12633731; doi:10.1242/jeb.250474)
Supplement: Dataset 6. Code instructions. [file jexbio-228-250474-Dataset6.zip › Liang_etal_2025_README.pdf]

README: statistical analysis from Liang et al., 2025

Data can be found in "tidyDataFiles"

Variables are described in brief below. Details can be found in text.

TidyDataSet_EBD

Fish: identity code for each fish
Size: size category for each fish (sizes listed in manuscript)
Treatment: treatment category for each fish
Experiment: numerical code for the group for each fish (details in manuscript)
Distance Along Fin (proximal to distal; %): distance along the fin lobe for each slice
Abductor Integrated Density: average integrated density in the Abductor for each slice
Adductor Integrated Density: average integrated density in the Adductor for each slice
Coracometapterygialis Integrated Density: average integrated density in the Coracometapterygialis for each slice
Zonopropterygialis Integrated Density: average integrated density in the Zonopropterygialis for each slice

TidyDataSet_emg

Fish: identity code for each fish
Trial: trial number for the value
Behaviour: identifier for whether the recorded value is during swimming or walking
Muscle: identifier for which muscle the value is from
Fin EMG Duration (full cycle; s): muscle activity duration during a locomotor cycle
Fin EMG Duty Factor (% full cycle): muscle activity duty factor during a locomotor cycle
Fin EMG Maximum Amplitude (full cycle; % max): muscle activity maximum amplitude during a locomotor cycle
Fin EMG RIA (full cycle; % max): muscle activity rectified integrated area during a locomotor cycle
Fin EMG Number of Bursts (per full cycle): number of muscle activity bursts during a locomotor cycle
Fin EMG Duration (propulsive phase; s): muscle activity duration during a propulsive phase
Fin EMG Duty Factor (% propulsive phase): muscle activity duty factor during a propulsive phase
Fin EMG Maximum Amplitude (propulsive phase; % max): muscle activity maximum amplitude during a propulsive phase
Fin EMG RIA (propulsive phase; % max): muscle activity rectified integrated area during a propulsive phase
Fin EMG Number of Bursts (per propulsive phase): number of muscle activity bursts during a propulsive phase
Fin EMG Duration (recovery phase; s): muscle activity duration during a recovery phase
Fin EMG Duty Factor (% recovery phase): muscle activity duty factor during a recovery phase
Fin EMG Maximum Amplitude (recovery phase; % max): muscle activity maximum amplitude during a recovery phase
Fin EMG RIA (recovery phase; % max): muscle activity rectified integrated area during a recovery phase

Fin EMG Number of Bursts (per recovery phase): number of muscle activity bursts during a recovery phase

TidyDataSet_kine

Fish: identity code for each fish
Trial: trial number for the value
Behaviour: identifier for whether the recorded value is during swimming or walking
Speed over ground (BL/s): the speed over ground recorded during a locomotor trial
Caudal fin swing distance (BL): swing distance of the caudal fin during a locomotor cycle
Pectoral fin swing distance (BL): swing distance of the pectoral fin during a locomotor cycle
Maximum pectoral fin elevation (BL): maximum elevation of the pectoral fin during a locomotor cycle
Minimum pectoral fin elevation (BL): minimum elevation of the pectoral fin during a locomotor cycle
Pectoral fin elevation range (BL): pectoral fin elevation range during a locomotor cycle
Maximum pectoral fin adduction (deg): maximum angle of adduction for the pectoral fin during a locomotor cycle
Minimum pectoral fin adduction (deg): minimum angle of adduction for the pectoral fin during a locomotor cycle
Pectoral fin adduction range (deg): range of pectoral fin adduction during a locomotor cycle
Maximum abduction velocity (deg/s): maximum velocity during abduction
Routine abduction velocity (deg/s): routine velocity during abduction
Maximum adduction velocity (deg/s): maximum velocity during adduction
Routine adduction velocity (deg/s): routine velocity during adduction
Nose swing distance (BL): swing distance of the nose for locomotor cycles during walking

Statistical Analysis can be found in "tidyLinearAnalysis.R"

This R code contains statistical analysis, data figures, and tables from Liang et al., 2025. Headings and comments throughout describe what each section does. The R Project file "Liang_etal_2025_RProject" can be loaded before running the R script to set the base directory to the folder that contains the R Project (this should mean that all file paths included in the script work without any changes required).
